# Silencing an *N*-Acyltransferase-Like Involved in Lignin Biosynthesis in *Nicotiana attenuata* Dramatically Alters Herbivory-Induced Phenolamide Metabolism

**DOI:** 10.1371/journal.pone.0062336

**Published:** 2013-05-21

**Authors:** Emmanuel Gaquerel, Hemlata Kotkar, Nawaporn Onkokesung, Ivan Galis, Ian T. Baldwin

**Affiliations:** 1 Department of Molecular Ecology, Max Planck Institute for Chemical Ecology, Jena, Germany; 2 Plant Molecular Biology Unit, Division of Biochemical Sciences, National Chemical Laboratory (CSIR), Pune, India; 3 Institute of Plant Science and Resources, Okayama University, Kurashiki, Japan; Friedrich-Alexander-University Erlangen-Nurenberg, Germany

## Abstract

In a transcriptomic screen of *Manduca sexta*-induced *N-acyltransferases* in leaves of *Nicotiana attenuata*, we identified an *N-acyltransferase* gene sharing a high similarity with the tobacco lignin-biosynthetic *hydroxycinnamoyl-CoA:shikimate/quinate hydroxycinnamoyl transferase* (*HCT*) gene whose expression is controlled by MYB8, a transcription factor that regulates the production of phenylpropanoid polyamine conjugates (phenolamides, PAs). To evaluate the involvement of this *HCT*-like gene in lignin production as well as the resulting crosstalk with PA metabolism during insect herbivory, we transiently silenced (by VIGs) the expression of this gene and performed non-targeted (UHPLC-ESI/TOF-MS) metabolomics analyses. In agreement with a conserved function of *N. attenuata HCT*-like in lignin biogenesis, *HCT*-silenced plants developed weak, soft stems with greatly reduced lignin contents. Metabolic profiling demonstrated large shifts (up to 12% deregulation in total extracted ions in insect-attacked leaves) due to a large diversion of activated coumaric acid units into the production of developmentally and herbivory-induced coumaroyl-containing PAs (*N*′,*N*′′-dicoumaroylspermidine, *N*′,*N*′′-coumaroylputrescine, etc) and to minor increases in the most abundant free phenolics (chlorogenic and cryptochlorogenic acids), all without altering the production of well characterized herbivory-responsive caffeoyl- and feruloyl-based putrescine and spermidine PAs. These data are consistent with a strong metabolic tension, exacerbated during herbivory, over the allocation of coumaroyl-CoA units among lignin and unusual coumaroyl-containing PAs, and rule out a role for HCT-LIKE in tuning the herbivory-induced accumulation of other PAs. Additionally, these results are consistent with a role for lignification as an induced anti-herbivore defense.

## Introduction

Plants as sessile organisms are exposed during their development to variable stress conditions which have shaped, from a macro-evolutionary point of view, the emergence of highly adaptive tolerance and resistance traits. The feeding damage of herbivores has provided a major evolutionary selective pressure that has sculpted all aspects of plant metabolism including the composition and size of their pools and the regulatory networks that determine fluxes [1]–[3]. When chewing leaves, insects elicit a burst of the plant hormone, jasmonic acid (JA); this signaling molecule mediates rapid changes in secondary metabolic pathways [4], [5]. Native tobacco plants, *Nicotiana attenuata,* accumulate, as a consequence of JA signaling, large amounts of nicotine, a neurotoxic compound and a spectrum of phenolic amide conjugates (phenolamides, PAs), derived from the phenylpropanoid pathway, to defend against specialist and generalist chewing herbivores [6], [7].

**Figure 1:**
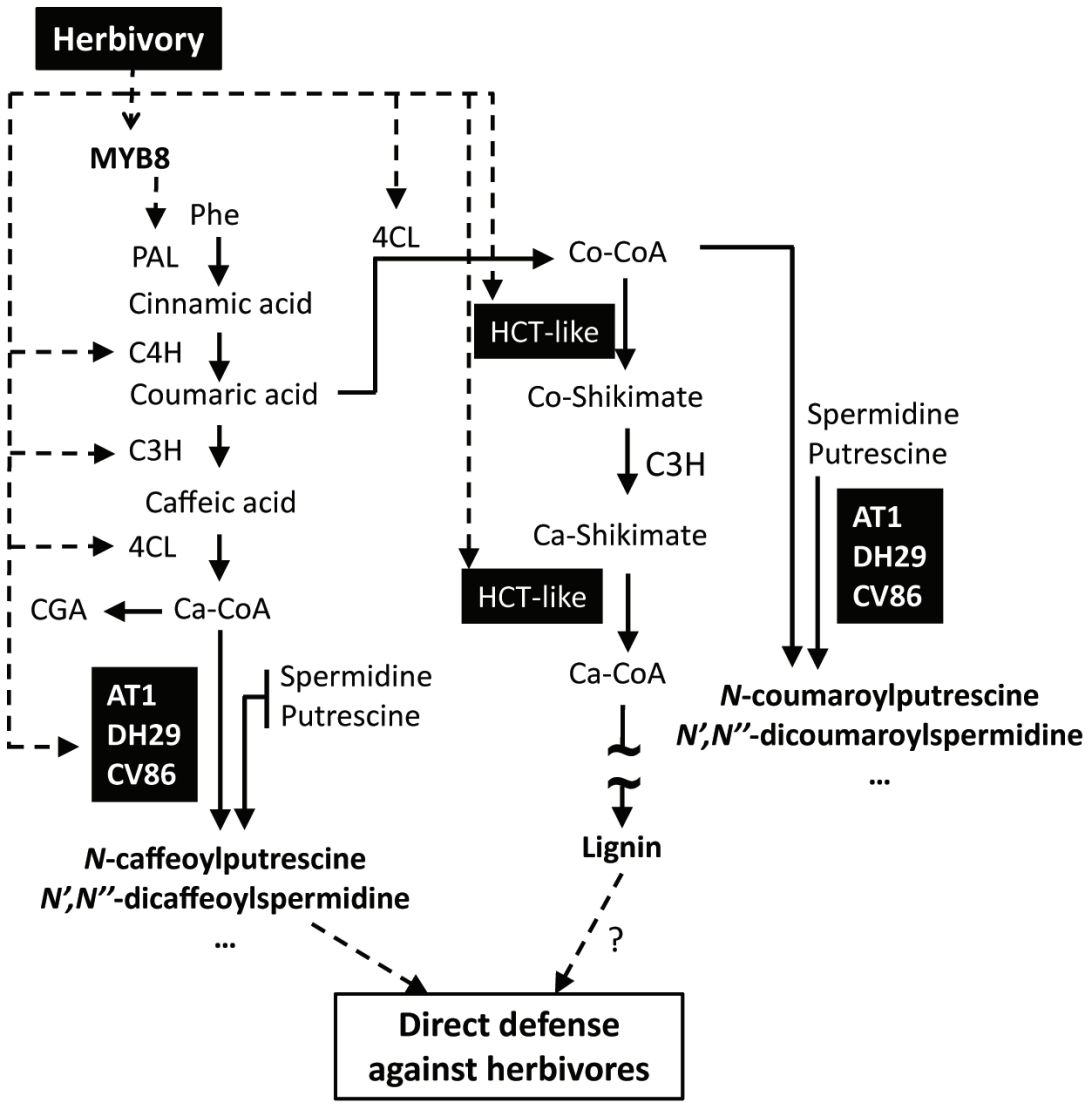
Proposed model of MYB8-regulated processes in *N.* attenuata plants. The MYB8 transcription factor is up-regulated in response to herbivory and activates the transcription of downstream genes in the phenylpropanoid pathway (PAL, C4H, C3H and 4CL, [6], [32]). MYB8-activated *N-*hydroxycinnamoyl acyltransferases lead to the accumulation of direct defense metabolites including *N*-caffeoylputrescine (AT1) and *N*′,*N*′′-dicaffeoylspermidine (DH29 and CV86) in attacked and systemic leaves of *N. attenuata* [9]. Simultaneously, MYB8 also activates the lignin-related acyltransferase HCT-LIKE (this report) which putatively promotes local and systemic lignification and mechanical defense. Enzymatic step are shown by solid lines; experimentally determined MYB8 regulatory steps are indicated by dashed lines.

As is frequently observed in transcriptomic analyses, herbivore-induced changes in a plant's metabolome result from specific reorganizations of metabolic pathways. However, how attacked cells rechannel metabolic fluxes towards the production of a specific spectrum of metabolites is frequently unknown. The underlying mechanisms by which plants perceive insect herbivory are known to be controlled by elicitors present in the oral secretions (OS) of feeding larvae [8]. As an essential part of the signal transduction mechanisms, plants employ specific transcription factors (TFs) to synchronize the expression of relevant gene networks. For example, genes controlling PA biogenesis in *N. attenuata* are tightly regulated by MYB8, an herbivory-inducible TF of the MYB family [6]. Figure 1 summarizes major steps in the formation of PA and connections with the lignin pathway. MYB8 activates the core phenylpropanoid genes and specific *N*-acyltransferases that redirect the flux of phenylpropanoids towards PA production during insect herbivory. Using microarray analysis and metabolic profiling of *MYB8*-silenced plants (irMYB8), we recently identified several *N*-acyltranferase enzymes that conjugate activated phenolic acids (*p-*coumaroyl-, caffeoyl-, feruloyl) with polyamines (putrescine and spermidine) [9]: AT1 is a hydroxycinnamoyl-CoA: putrescine acyltransferase responsible for caffeoyl- and coumaroyputrescine accumulation during insect herbivory. Another gene (DH29), specific for spermidine conjugation, mediates the initial acylation step in the formation of caffeoyl-, coumaroyl and feruloyl-containing di-acylated spermidine structures. Although this enzyme was not able to perform the second acylation towards diacylated spermidine biosynthesis, another acyltransferase gene, called CV86, proposed to act on mono-acylated spermidines was isolated and partially characterized [9].

Among the herbivory-regulated genes screened in the aforementioned study, we identified a candidate *N*-acyltransferase which shared a high sequence similarity with previously characterized *hydroxycinnamoyl-CoA:shikimate/quinate hydroxycinnamoyl transferase* (*HCT*) genes from *N. tabacum* (Acc. No. CAD47830) and *N. benthamiana* (Acc. No. CAD88491) (Figure S1). HCT enzymes have previously been shown to catalyze developmentally-regulated lignin biosynthesis in tobacco [10], [11]. Interestingly, the herbivory-induced expression of this *HCT-like* gene is dependent on and is temporally synchronized by MYB8 transcriptional activity. This suggests that MYB8 controls, via an HCT-like conserved activity, certain aspects of lignin production in herbivore-attacked leaf tissues. The possibility of cross-talk between HCT activity and the PA pathway (Figure 1), especially when the PA pathway is maximally induced during insect herbivory [9] has not been rigorously examined.

To dissect the metabolic processes controlled by HCT-like activity and its potential competition with PA metabolism during insect herbivory, we silenced the expression of this gene using a virus-induced gene silencing (VIGs) procedure optimized for this plant species. The VIGs approach allows genes to be silenced in developed seedlings with normal morphologies, thus avoiding the confounding effects of developmental alterations on metabolism that are commonly observed when essential genes are stably silenced [12], [13]. While HCT-like apparently functions *in planta* as an HCT enzyme required for lignin deposition, we also uncovered a strong rerouting of the flux of activated coumaric acid units towards the production of developmentally and herbivory-induced coumaroyl-containing PAs, and to a lesser degree, towards phenolic accumulation.

## Materials and Methods

### Plant material and growth conditions

Wild-type *Nicotiana attenuata* Torr. ex Watson from an inbred line in its 31^st^ generation were used for all experiments. The seeds were collected from plants grown on private lands (DI ranch in southwestern Utah, USA) and since *N. attenuata* is not an endangered plant species, no collection permits were necessary. The owner of the private land at the time of the collections gave permission to conduct the collections. Seeds were treated for 1 h with diluted smoke and 0.1 µM GA_3_ solution and placed on sterile Gamborg's B5 agar medium in petri dishes. Seedlings were maintained at 26°C/16 h, 155 μmol m^−2^ s^−1^ light: 24°C/8 h dark cycle in the growth cabinet for 10 days and subsequently transplanted to a peat-based substrate in small Teku plastic pots in the glasshouse. Plants were maintained at day/night cycle of 16 (26–28°C)/8 (22–24°C) h under supplemental light from 600 W high-pressure sodium lamps (Philips Sun-T Agro). After 10 d in the glasshouse, the plantlets were transferred to 1 L pots with soil and placed in a growth chamber under the same light conditions but at 20°C. Plants were kept in the growth chamber under constant humidity ∼65% and the low temperature regime (20°C) for the rest of the experiment.

### VIGs procedure

A virus-induced gene silencing (VIGs) system based on the tobacco rattle virus (TRV) was used as described by (Saedler and Baldwin, 2004). A 167 bp fragment of the *acyltransferase2* (*HCT-LIKE*) gene was PCR-amplified using a primer pair shown in Figure S2. A gel-purified *HCT-LIKE* fragment was cloned into *Bam*HI-*Sal*I sites of the polylinker in the pTV00 vector to obtain pTV-HCT-LIKE. Correct plasmid clones were transformed by electroporation into the *Agrobacterium tumefaciens* strain GV3101: pTV-HCT-LIKE for *HCT-LIKE* gene silencing, pTV00 plasmid without insert (empty vector; EV) as a negative control and pTV-PD, a *phytoene desaturase-*silencing construct (pTV-PD) as a positive control. Three leaves of 24–25 day-old *N. attenuata* plants were infiltrated with a 1∶1 mixture of *A. tumefaciens* strains carrying pBINTRA vector and one of the pTV-HCT-LIKE, pTV00 (EV) or pTV-PD constructs. VIGs progression was monitored in a set of inoculated plants with pTV-PD which bleaches leaves due to the depletion of carotenoids. Experiments on VIGs-HCT-LIKE and VIGs-EV plant treatments were initialized after the pTV-PD-inoculated leaves developed the distinct bleaching phenotype demonstrating that the gene silencing process was well underway. 200 bp fragments of the gene to be silenced guarantee effective gene silencing. In general, 23 nt of identity between the fragment and the target gene is sufficient for direct post-transcriptional silencing of a gene. To achieve single gene silencing (i.e to avoid post-transcriptional silencing of closely related gene sequences), sequence identity of more than 22 nt with other genes has to be avoided, and this is usually accomplished by selecting sequences in the un-transcribed regions of the gene. This procedure was strictly followed for selecting the DNA sequence used for HCT-like VIGs-silencing. To rule out the possibility of off-target silencing, the HCT-like DNA fragment used for VIGs was blasted against a full transcriptome database obtained by 454-sequencing to ensure that this fragment did not have a sequence identity of more than 22 nt with other genes.

### Microscopy

Stems of elongated plants were hand-sectioned with a razor blade at the base of the plant (1 cm above the ground) and stained with 0.05% Toluidine blue O to visualize lignified cell walls. After a brief rinsing of the cross-sections in distilled water, the samples were mounted on microscope slides and observed in bright field with a Leica DM 6000B light microscopy system equipped with a CCD camera HV-D20P (Hitachi Kokusai Electric Inc., Tokyo, Japan). Photographs were processed with a LM Image Manager (Leica Microsystems). Images were annotated using the Power Point software from the Microsoft Office 2010 package.

### Growth parameters

VIGs-EV and VIGs-HCT-LIKE plant growth parameters, i.e. stalk length and diameter (1 cm and 20 cm above the base), were determined at the end of the experiment.

### qRT-PCR analysis

Trizol reagent (Invitrogen; http://www.invitrogen.com) was used to extract total RNA. Approximately 150 mg of powdered leaf material ground in liquid nitrogen was extracted following manufacturer's protocol. Crude RNA samples were treated with RQ1 DNase (Promega; http://www.promega.com), followed by phenol/chloroform/isoamylalcohol (25∶24∶1) extraction and ethanol precipitation. DNA-free RNA samples (500 ng) were reverse-transcribed using oligo (dT_18_) primers and Superscript II enzyme (Invitrogen) following the manufacturer's recommendations. All qRT-PCR assays were performed with a Stratagene MX3005P instrument (Stratagene; http://www.stratagene.com) using the parameters recommended by the manufacturer. Expression values of the *Elongation factor-1α* gene (*EF1-α*, Acc. No. D63396) were used for normalization of transcript levels. Gene-specific primers located outside of the silencing region (*HCT-LIKE* forward: 5′-AGACTGTGTAGGGACGAGGATGG-3′, *HCT-LIKE* reverse: 5′-AGTGAAGACCAGAAGCTCCATCTG-3′) was used for SYBR Green-based determination of *HCT-LIKE* transcript levels.

### Targeted analysis of secondary metabolites by HPLC-PDA

High performance liquid chromatography coupled to photodiode array detection (HPLC-PDA) was used for the quantification of secondary metabolites. Samples (approximately 100 mg) were ground in liquid nitrogen and extracted by adding 1 mL of acidified 40% MeOH, prepared with 0.5% (v/v) acetic acid/water, to each sample in 2 mL microcentrifuge tubes with metal balls. Samples were homogenized in the ball mill (Genogrinder 2000, SPEX CertiPrep, Metuchen, New Jersey, USA) for 45 sec at 250 strokes per min and centrifuged at 16,000 *g*, 4°C for 30 minutes. Supernatants were transferred into 1.5 mL microcentrifuge tubes, re-centrifuged under the same conditions and finally transferred into 2 mL glass vials before analysis on the Agilent-HPLC 1100 series (http://www.chem.agilent.com). One µL of the sample was separated with a Chromolith FastGradient RP 18-e column (50×2 mm, monolithic silica with bimodal pore structure, macropores with 1.6 µm diameter, Merck, Darmstadt, Germany) connected to a pre-column (Gemini NX RP18, 2 x 4.6 mm, 3 µm). Mobile phases –0.1% formic acid +0.1% ammonium water, pH 3.5 as solvent (A) and methanol as solvent (B) – were used in a gradient mode with the following conditions: time/concentration (min/%) for B: 0.0/0, 0.5/0, 6.5/80, 9.5/80 and reconditioning for 5 min to 0% B. The flow rate was 0.8 mL/min and column oven temperature was set to 40°C. Levels of dicaffeoylspermidine (DCS), caffeoylputrescine (CP), chlorogenic acid (CGA), crypto-chlorogenic acid (crypto-CGA) and rutin were determined as described previously [6]. Calibration curves generated by injecting increasing concentrations of authentic standards were used for the calculation of nicotine, CGA and rutin concentrations. Levels of DCS isomers, crypto-CGA and CP were expressed as CGA equivalents, since no authentic standards were available for these compounds.

### UPLC/ESI-TOF-MS metabolomics measurements

To analyze the breadth of *N. attenuata* phenolic-based metabolism, 2 µL of the leaf extracts prepared as above were separated using a Dionex RSLC system (Dionex, Sunnyvale, USA) equipped with Dionex Acclaim 2.2 μm 120A 2.1×150 mm column, applying either a short separation binary gradient (flow rate 300 µL min^−1^) with the following parameters, 0 to 0.5 min isocratic 80% A (deionized water, 0.1% [v/v] acetonitrile [Baker, HPLC grade] and 0.05% formic acid), 20%B (acetonitrile, 0.05% formic acid); 0.5 to 2 min linear gradient to 40% B; 2 to 6 min isocratic 40% B, 6 to 10 min linear gradient to 80% B or a long separation gradient optimized for phenolamides (flow rate 300 µL min^−1^) 0 to 5 min isocratic 95% A, 5% B; 5 to 20 min linear gradient to 32% B; 20 to 22 min linear gradient to 80% B; isocratic for 6 min. Eluted compounds were detected by a MicroToF mass spectrometer (Bruker Daltonics, Bremen, Germany) equipped with an electrospray ionization source in positive ionization mode as previously described [15]. Typical instrument settings were as follows: capillary voltage 4500 V, capillary exit 130 V, dry gas temperature 200°C and dry gas flow of 8 L min^–1^. Ions were detected from m/z 200 to 1400 at a repetition rate of 1Hz. Mass calibration was performed using sodium formate clusters (10 mM solution of NaOH in 50/50% v/v isopropanol/water containing 0.2% formic acid).

### Non-targeted comparative processing of UHPLC/ESI-TOFMS data

Raw data files were converted to netCDF format using the export function of the Data Analysis version 4.0 software (Bruker Daltonics, Bremen, Germany) and processed using the XCMS package (http://metlin.scripps.edu/download/). Peak detection was performed using the ‘centWave’ method and parameter settings ppm  = 20, snthresh  = 10, peakwidth =  c(5,18). Retention time correction was achieved using the parameter settings minfrac  = 1, bw  = 10 s, mzwid  = 0.1 D, span  = 1, missing  =  extra  = 0. After peak grouping and filling in of missing features using the fillPeaks routine of the XCMS package [14], the obtained data matrix was imported into Microsoft Excel for statistical analysis. Ion traces were deconvoluted and putative in-source pseudo-spectra reconstructed with the R package CAMERA (http://www.bioconductor.org/packages/release/bioc/html/CAMERA.html) with defaults parameters. Isotopic peaks and multiple-charged *m/z* signals detected by CAMERA were excluded to reduce the redundancy within the data matrix. Consistent mass features, which were at least present – for a single factorial group – in four out of the five biological replicates with Rt >1 min were considered for further analysis. Zero values remaining after applying the ‘filling in’ function in XCMS were replaced by half of the minimum positive value of the row in the original data.

### Phenolamide annotation and identification

Identifications and annotations of *N. attenuata* phenolic derivatives are based on MS/MS CID-induced fragmentation experiments performed on a MaXis q-TOFMS and *in vivo N*-containing fragment labeling with ^15^KNO_3_ for high precision diagnostic fragment elemental composition assignment [15]. Original data are reported in [15] and examples presented as File S1. Structural rearrangements during in source ionization and fragmentation did not allow for the unequivocal assignment of the phenylpropanoid residues to the different *N* positions of putrescine and spermidine backbones. *N*-caffeoylputrescine, *N*-feruloylputrescine and *N*-coumaroylputrescine were unambiguously identified based on comparison of obtained mass spectral data and retention times with those of synthetic standards. Numbers in the compound name column of Table 1 refer to the different annotation levels defined by the Metabolomics Standard Initiative [16].

**Table 1:** Abundant coumaroyl-based PAs measured by UPLC-TOFMS in methanol-water leaf extracts of *N. attenuata* VIGs plants.

Label	Rt (s)	Precursor *m/z*	Ion type	Elemental Formula	Error (ppm)	Ab.	Annotation	W+OS				Caterpillar		
								*P-value*			FC	*P*		FC
**Co1**	208	*m/z* 292.202	[M+H]^+^	C_16_H_26_N_3_O_2_^+^	1.1	MCoS	*^2^N*-Coumaroylspermidine isomer#1	0.000		***	73.3	0.000	***	22.3
**Co2**	272	*m/z* 292.201	[M+H]^+^	C_16_H_26_N_3_O_2_^+^	1.8	MCoS	*^2^N*-Coumaroylspermidine isomer#2	0.000		***	66.8	0.000	***	21.4
**Co3**	326	*m/z* 235.142	[M+H]^+^	C_13_H_19_N_2_O_2_^+^	1.7	CoP	*^2^N*-Coumaroylputrescine isomer#1	0.000		***	258.4	0.000	***	10.8
**Co4**	395	*m/z* 235.142	[M+H]^+^	C_13_H_19_N_2_O_2_^+^	1.7	CoP	*^2^N*-Coumaroylputrescine isomer#2	0.000		***	43.5	0.000	***	8.3
**Co5**	438	*m/z* 452.219	[M+H]^+^	C_25_H_30_N_3_O_5_^+^	0.8	-	Unknown	0.001		**	9.9	0.000	***	15.3
**Co9**	614	*m/z* 458.264	[M+H]^+^	C_25_H_36_N_3_O_5_^+^	0.9	-	Unknown	0.000		***	26.9	0.000	***	124.0
**Co10**	647	*m/z* 454.250	[M+H]^+^	C_25_H_32_N_3_O_5_^+^	0.6	CoCS	*^2^N′,N′′-*Coumaroyl,caffeoylspermidine isomer#1	0.000		***	41.0	0.001	**	27.3
**Co11**	678	*m/z* 456.263	[M+H]^+^	C_25_H_34_N_3_O_5_^+^	0.6	-	*^3^N′,N′′-*Monohydrated coumaroyl,caffeoylspermidine isomer#3*	0.000		***	89.1	0.000	***	22.3
**Co12**	690	*m/z* 454.250	[M+H]^+^	C_25_H_32_N_3_O_5_^+^	0.6	CoCS	*^2^N′,N′′-*Coumaroyl,caffeoylspermidine isomer#3	0.000		***	31.3	0.000	***	18.5
**Co13**	721	*m/z* 454.250	[M+H]^+^	C_25_H_32_N_3_O_5_^+^	0.6	CoCS	*^2^N′,N′′-*Coumaroyl,caffeoylspermidine isomer#4	0.000		***	23.7	0.000	***	10.4
**Co14**	734	*m/z* 438.253	[M+H]^+^	C_25_H_32_N_3_O_4_^+^	0.2	CoCoS	*^2^N′,N′′-*Di*-*coumaroylspermidine isomer#1	0.025		*	0.9	0.005	**	6.3
**Co15**	756	*m/z* 440.255	[M+H]^+^	C_25_H_34_N_3_O_4_^+^	0.9	-	*^3^N′,N′′-*Monohydrated Coumaroyl,caffeoylspermidine isomer#2	0.025		*	0.9	0.011	*	4.2
**Co16**	777	*m/z* 438.252	[M+H]^+^	C_25_H_32_N_3_O_4_^+^	0.3	CoCoS	*^2^N′,N′′-*Di*-*coumaroylspermidine isomer#4	0.000		***	2.7	0.000	***	102.5

Elemental formulas and relative mass errors (in ppm) were calculated using Smart Formula from the UPLC-TOFMS operating software (see Method section). Candidate formulas were ranked according to both mass deviation and isotope pattern accuracy reflected in the sigma value. MS/MS+ spectra for some of the reported parent ion and the strategy used for compound annotation are summarized in Files S1 and S2 and characteristic ion fragments reported in [9], [15] were used for compound annotation. Asteriks indicate significant changes between VIGs-HCT-LIKE and VIGs-EV samples in the relative intensity of reported metabolites. Numbers in the compound name column refer to the different annotation levels defined by the Metabolomics Standard Initiative. Ab, abbreviation; FC, fold-changes (VIGs-HCT-LIKE>VIGs-EV); Rt, retention time.

### Statistical analysis

For principal component analysis (PCA) of UHPLC/ESI-TOFMS profiling data, we first filtered the data in order to identify and remove *m/z* signals that are unlikely to be of use when modeling the data. No phenotype information was used during the filtering process and original *m/z* features that were considered as near-constant throughout the experiment conditions based on their coefficient of variation (mean divided by standard deviation) were removed. We used the Pareto scaling – mean-centered and divided by the square root of the standard deviation of each variable – method for data normalization. The PCA analysis was performed using the ‘prcomp’ package for R via the MetaboAnalyst interface [17]. The HPLC data and RT-qPCR data were analyzed with PASW statistic 18 (SPSS) software.

## Results and Discussion

### *HCT-LIKE* is a *MYB8*-controlled and herbivory-induced *N*-acyltransferase gene

**Figure 2:**
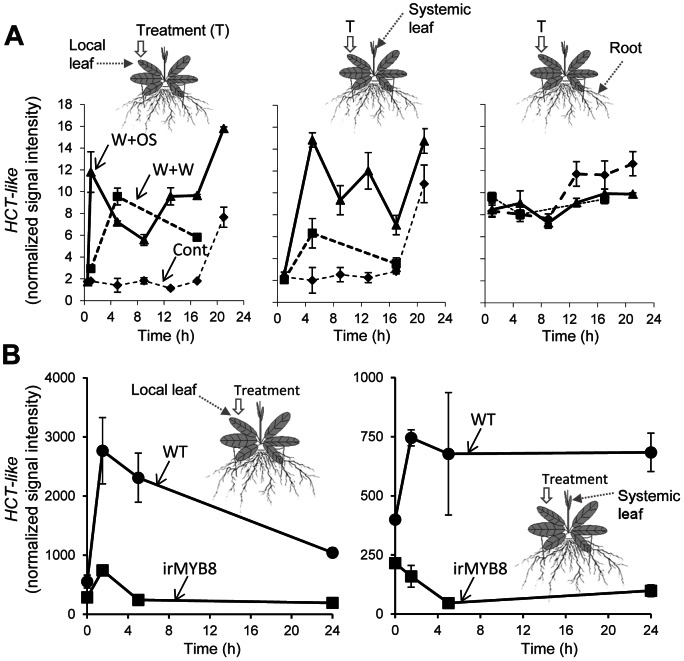
Herbivory-induced and MYB8-dependent expression of *Nicotiana attenuata HCT-LIKE*. (**A**) Local (treated leaf) and systemic (systemic unwounded leaves and roots) expression of *HCT-LIKE* gene in wild-type (WT) plants was determined by microarray analysis using wounding (W+W) and simulated herbivory (W+OS) elicited plants. Control plants remained untreated. (**B**) Local and systemic down-regulation of *HCT-LIKE* expression in *MYB8*-silenced plants (irMYB8). Values are means of three replicate measurements ± SE.

A search of public databases containing tobacco expressed sequence tags (ESTs) yielded a large number of candidate *N*-acyltransferase genes classified into the BAHD protein superfamily. In an earlier study [9], we analyzed the expression patterns of these genes using a microarray system designed for *N. attenuata* (data-set publicly available at GEO identifier GPL13527) and discovered that several of these genes shared temporal and tissue-specific and patterns of expression with that of *MYB8*, a phenolamide (PA)-specific transcription factor (TF). The candidate *N*-acyltransferase gene was not only co-regulated with *MYB8* but it also showed a strongly down-regulated pattern of expression in elicited irMYB8 leaves (Figure 2), consistent with its inclusion in the *MYB8*-regulated gene network presented in Figure 1. Interestingly, the deduced protein sequence of this *N*-acyltransferase showed high similarity to the previously characterized hydroxycinnamoyl-CoA:shikimate/quinate hydroxycinnamoyl transferase (HCT) enzymes from *N. tabacum* and *N. benthamiana* (Figure S1) which participates in lignin biosynthesis [10], [11]. Hence the homology-predicted function of this *N*-acytransferase contrasted with its expected function in PA biosynthesis based on its MYB8-dependent regulation.

To understand the influence of HCT-LIKE on PA and lignin biogenesis in *N. attenuata*, we transiently silenced the expression of this *HCT-LIKE* gene via virus-induced gene silencing (VIGS). The VIGs procedure has been extensively used to identify the function of key *N. attenuata* metabolic genes, including a recent study on PA biosynthetic genes [9]. This transient gene-silencing procedure has the distinct advantage over constitutive gene silencing procedures of avoiding severe developmental alterations that compromise the conclusions about the metabolic function of particular genes. As expected, transcripts levels of HCT-LIKE were reduced by approximately 90% in leaves of *N. attenuata* VIGs-HCT-LIKE plants, both in unwounded leaves as well as in those on which *Manduca sexta* larval attack was simulated by applying oral secretions to mechanical wounds (W+OS) (Figure S2).

### Silencing *HCT-LIKE* affects lignin deposition and stem morphology

**Figure 3:**
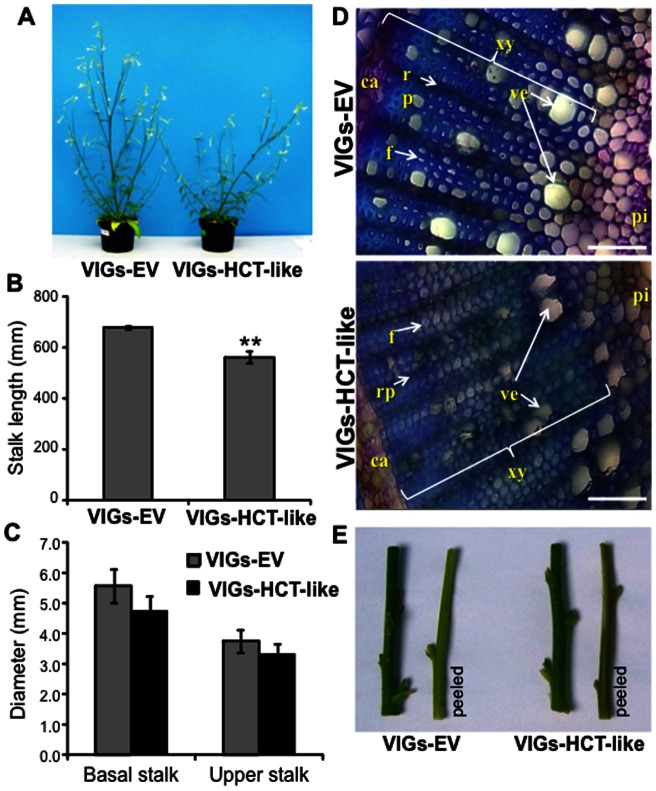
Growth, stem ultrastructure and reduced lignin deposition of VIGs-HCT-LIKE compared to that of VIGs-EV plants. (**A**) *HCT-LIKE*-silenced plants have strongly altered morphology with reduced stalk lengths and leaning “rubbery” stem phenotypes. (**B**) Stalk length and (**C**) basal and upper stalk (1 and 20 cm, respectively from the base of the stem) diameters of VIGS plants (n = 5 biological replicates) measured approximately 40 d after VIGS inoculation of plants (** P<0.01, Student's *t*-test). (**D**) High lignin deposition in EV (empty vector) stem sections as compared to in *HCT-LIKE*-silenced (VIGs-HCT-LIKE) stems. Observation made under bright field microscopy conditions after staining of hand sections with Toluidine blue O. ca, cambium; xy, xylem; rp, ray parenchyma; f, xylem fiber; ve, vessel; pi, pith. Bars  = 100 μm. (E) Intact and peeled stem segments of VIGs-EV and VIGs-HCT-like plants.

Silencing *HCT-LIKE* (VIGs-HCT-LIKE) strongly affected the plant's morphology, producing smaller plants with leaning, weak soft stems compared to VIGS-EV plants (Figure 3A). While stalk lengths, measured 40 days after VIGs inoculation, was significantly reduced in VIGs-HCT-LIKE compared to VIGs-EV plants (Figure 3B), the lower and upper stem diameters measured 1 and 20 cm from the base of the rosette were reduced in VIGs-HCT-LIKE, although these differences were not statistically significant (Figure 3C). In agreement with the high sequence conservation between *HCT-LIKE* and the *HCT* genes involved in lignin biosynthesis [10], [11], the growth phenotype in VIGs-HCT-LIKE plants suggested that HCT-LIKE functions as an HCT enzyme in *N. attenuata*.

To further examine alterations in the lignification of VIGs-HCT-LIKE plants, we analyzed the microscopic lignin deposition patterns in these as well as in VIGs-EV control plants. Lignin is a major component of the cell wall that provides mechanical strength and impermeability to vascular tissues. Chemically, lignin is a phenolic heteropolymer derived from hydroxycinnamoyl alcohols, mainly *p-*coumaryl alcohol, coniferyl alcohol and sinapyl alcohol, through a free radical coupling reaction [18]. Lignin deposition can be rapidly assessed by a polychromatic staining of the cell walls by Toluidine blue O staining [19]. Staining of the hand-cut cross-sections of the stem 1 cm above the base of the plants' rosette with a 0.05% [w/v] solution of Toluidine blue O in 0.1 M phosphate buffer (pH 6.8) revealed strongly decreased lignification which resulted in thin irregular cell walls in the xylem vasculature of *HCT-LIKE*-silenced plants (Figure 3D). Additionally, a slightly red-brown color was observed in the xylem of peeled stem segments of VIGs-HCT-like plants (Figure 3E). The altered xylem morphology determined from the inspection of cross-sections of stems was comparable to that observed in *HCT*-silenced *N. benthamiana* plants, changes which are known to be due to reduced lignin deposition [10]. In contrast, VIGs-EV plants showed strong and stable stems with thick-walled xylem cells, confirming that the VIGs procedure itself did not interfere with lignin deposition (Figure 3D).

The generation, using the RNAi technology, of stably *HCT-LIKE*-silenced *N. attenuata* plants was also conducted. Not surprisingly, T_0_ transformed plants showed severely stunted growth (not shown). This stunted phenotype is consistent with observations made for *HCT* mutants [10] and has been functionally associated not only with reduced lignification but also with altered auxin transport due to flavonoid over-accumulation [20]. Recent studies have ruled out the role of flavonoids in the stunted phenotype of Arabidopsis *HCT* mutants and are consistent with the idea that a biochemical pathway downstream of coniferaldehyde is disrupted in *HCT* mutants [21]. These strong developmental alterations compromise the utility of these transgenic plants for comparative metabolic studies with wild type plants. In VIGs-HCT-LIKE plants, the new lignin produced after HCT-LIKE-silencing (induced at an advanced developmental stage) is added to the wild-type lignin produced before the VIGs procedure which likely explains why transient silencing of *HCT-LIKE* by VIGs did not result in stunted growth. Hence, comparative metabolomics experiments presented below were performed exclusively with VIGs-EV and VIGs-HCT-like plants.

No visible lignin deprivation-related phenotypes have been observed in plants stably silenced for MYB8 expression [6]. This suggests that *HCT-like* involvement in lignin biosynthesis is most likely regulated by an independent transcriptional activator during growth and development. Additionally, it remains to be determined whether MYB8′s role is direct or via a retro-route involving the flux of MYB8-dependent metabolites into lignin biosynthesis. Gene expression analyses of *N. attenuata* suggest an important role for lignification in the plant's response to herbivory. For example, transcripts of the lignin biosynthesis-related *cinnamyl alcohol dehydrogenase* (*CAD*) gene have been shown to accumulate in herbivory-elicited leaves of *N. attenuata* plants [22]. Consistent with a protective function of lignin, stem-boring weevil larvae have been shown under natural conditions to take advantage of the softer stems of lignin-deprived *N. attenuata* plants [23].

### *HCT-LIKE*-based lignin disruption results in pathway-specific deregulations of soluble phenolic derivatives

Major advances have recently been made in our understanding of the metabolic cross-talk within the downstream branches of the phenylpropanoid pathway by analyzing the impact of the silencing of lignin biosynthetic genes [18], [20], [24]–[27]. In tobacco, HCT catalyzes the formation of *p-*coumarate esters using shikimate or quinate units as acyl acceptors [11] and silencing leads to the constitutive redirection of this metabolic flux into flavonoid production through the activity of chalcone synthase enzymes [11], [20] but its influence on PA accumulation has not been examined. In *MYB8*-silenced *N. attenuata* plants, PA disruption strongly influenced the accumulation of multiple activated phenolic residues [9], indicating that the herbivory-induced increase in the production of PA results in a strong metabolic demand on the flux of phenylpropanoids. We therefore initially hypothesized that silencing *HCT-LIKE* would directly channel activated coumaric acid units towards conjugation with polyamine and that this flux may be modulated by the strength of the herbivory treatment inflicted to the leaves of VIGs plants.

**Figure 4:**
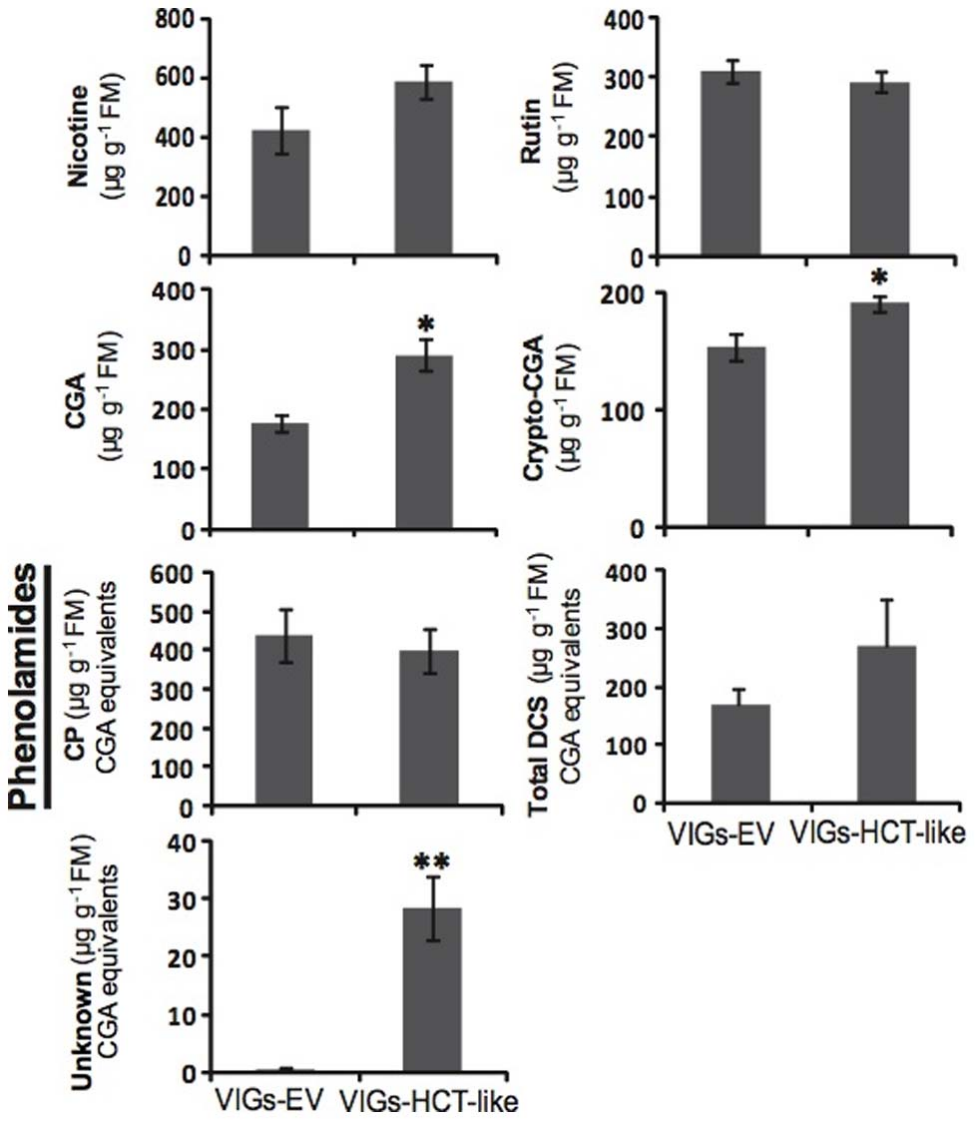
Nicotine and abundant phenolics in herbivory-elicited HCT-LIKE-silenced and EV plants. Levels (n = 5 biological replicates) of two pooled isomers of dicaffeoylspermidine (DCS), an unknown phenolamide, caffeoylputrescine (CP), chlorogenic acid (CGA), crypto-chlorogenic acid (crypto-CGA), rutin and nicotine were determined in leaves of an empty vector (EV) control and of *HCT-LIKE-*silenced *Nicotiana attenuata* after *Manduca sexta* attack. CP, DCS, crypto-CGA (expressed as CGA equivalents), CGA was determined at UV abs. 320 nm, rutin at 360 nm and nicotine at 260 nm. * P<0.05, ** P<0.01; Student's *t*-test.

We first performed routine HPLC-UV measurements of *Manduca sexta*-attacked VIGs-HCT-LIKE and VIGs-EV leaves (4 day feeding; Figure 4). As expected, and in agreement with HCT-LIKE-activated deregulations predicted to specifically influence phenylpropanoid derivatives, the accumulation of nicotine was unchanged in VIGs-HCT-LIKE plants. Consistent with the revealed compensatory response observed in *HCT*-silenced tobacco plants, chromatographic peaks corresponding to chlorogenic (CGA) and crypto-chlorogenic acids (CGA) were significantly increased by 40 to 50% in VIGs-HCT-LIKE compared to in EV plants. Additionally to *HCT*, the silencing of other genes downstream in the PAL pathway is known to increase CGA accumulations [10]. The increased CGA levels are consistent with the idea that HCT-LIKE, like HCT in tobacco plants, acts predominantly as a shikimate, rather than a quinate, conjugating enzyme. In contrast with reports made for constitutive levels of different flavonoid glycosides in *HCT*-silenced Arabidopsis plants [20], levels of rutin, the most abundant metabolite of this compound class in *N. attenuata* leaves, did not change in response to reduced *HCT-LIKE* transcript levels. Caffeoylputrescine (CP) and dicaffeoylspermidine (DCS) isomers, the most abundant PAs in *N. attenuata* leaves [28], were not significantly influenced by *HCT-LIKE* silencing. However, inspection of the UV-trace obtained from VIGs-HCT-LIKE plants revealed a peak corresponding to an unknown PA derivative and whose intensity was strongly amplified compared to VIGs-EV plants (Figure 4), motivating a higher resolution, MS-based analysis.

### Silencing *HCT-LIKE* diverts activated *p*-coumaric acids to coumaroyl-based PA production

To achieve a higher resolution in the characterization of *HCT-LIKE*-silenced plants, we performed metabolomics analyses by UHPLC-ESI/TOF-MS of leaves collected from uninduced (Ctrl), *M. sexta*-attacked and W+OS-treated VIGs plants. In a previous study [9], we demonstrated that direct *M. sexta* feeding resulted in more pronounced PA accumulations than did the W+OS treatment. A dramatic increase in the levels of mono-acylated spermidine conjugates, the first step in the biogenesis of poly-acylated derivatives, was observed compared to the almost undetectable levels of these metabolites after a single W+OS elicitation. Analyzing the metabolic responses elicited by these two treatments allowed us to capture the broadest picture of PA metabolism by taking advantage of the high temporal reproducibility afforded by the W+OS procedure and of the severe metabolic response that results from continuously chewing insect larvae.

**Figure 5:**
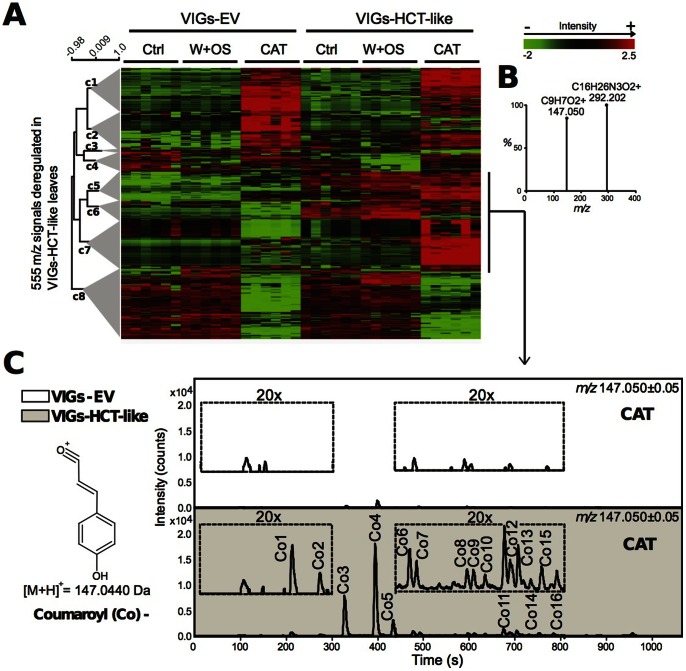
Silencing *HCT-LIKE* diverts activated *p*-coumaric acid to coumaroyl-containing PAs. (**A**) Hiearchical clustering analysis involving 555 deconvoluted *m/z* features differentially regulated (2-fold change, P-value <0.05) in leaves harvested from uninduced, *Manduca sexta*-attacked and W+OS-treated VIGs-HCT-LIKE compared to similarly treated VIGs-EV plants. (**B**) Many of the in-source generated pseudo-spectra reconstructed by CAMERA analysis for highly altered ion clusters (c5, c6 and c7) contained the typical signature fragments for coumaroyl moieties ([M+H]^+^ *m/z* 147.04±0.05) released after the cleavage of the ester bond linking them to a core molecule (sugar, polyamines, small acids, etc). (**C**) Representative ion chromatograms (n = 5 biological replicates) calculated for coumaroyl-containing metabolites in *M. sexta*-attacked leaves. “Co” numbers refer to main the PAs summarized in Table 1. Ctrl: uninduced control leaves; W+OS: mechanically wounded leaves treated with *M. sexta* oral secretions; CAT: *M. sexta* caterpillar-attacked leaves.

Deconvoluted metabolic profiles from both uninduced and elicited leaves of VIGs-HCT-LIKE plants were clearly separated from those from VIGs-EV plants in PCA projection plots (Figure S3) indicating larger deregulations than those originally described from the HPLC-UV traces. Consistent with the hypothesis that competition between PA and HCT-like-dependent metabolism is modulated by the strength of the herbivory stress inflicted, the proportion of 2-fold regulated ion signals (671 after herbivore attack vs 434 after W+OS treatment) was maximal in VIGs-HCT-LIKE herbivore attacked leaves (Figure S4). To better understand this stronger metabolic response, we performed a hierarchical clustering analysis for 555 *m/z* features influenced by *HCT-like* expression (File S2 for the complete peak matrix) and annotated the in-source pseudo-spectra present in clusters c5, c6, c7 in which the largest changes were detected (Figure 5). Many of the pseudo-spectra reconstructed using the CAMERA processing program contained the typical signature fragment of coumaroyl moieties ([M+H]^+^ *m/z* 147.04±0.05) released after the cleavage of the ester bond linking them to a core molecule (sugar, polyamine, small acid). We computed the trace for this *m/z* signal (Figure 5) as well as those of other phenolic residues – caffeoyl ([M+H]^ +^ *m/z* 163.04±0.05) and feruloyl moieties ([M+H]^ +^ *m/z* 177.04±0.05) (Figure S5). Table 1 summarizes the changes in hydroxycinnamoyl-containing PAs.

**Figure 6:**
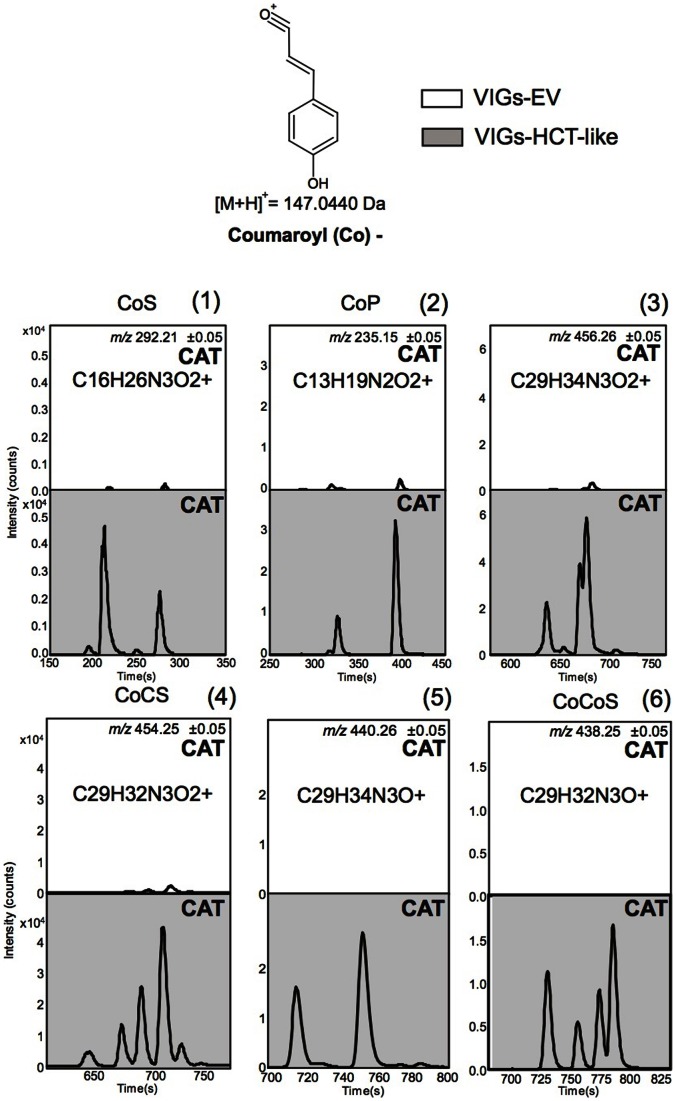
Precursor ions of abundant coumaroyl-containing PAs are up-regulated in response to *HCT-LIKE* silencing. Representative (n = 5 biological replicates) extracted ion chromatograms (EIC) for precursors of VIGs-HCT-LIKE-specific coumaroyl-based mono-acylated putrescines and mono- and di-acylated spermidines (Table 1). Ion types selected for the calculation of EIC traces correspond to the protonated [M+H]^+^ *m/z* signals of the different isomers of (1): *N*′-coumaroylspermidine (CoS), (2): *N*- coumaroylputrescine, (3): monhydrated *N*′,*N*′′- coumaroyl, caffeoylspermidine, (4): *N*′,*N*′′- coumaroyl, caffeoylspermidine (CoCS), (5): monohydrated *N*′, *N*′′-di-coumaroylspermidine and (6): *N*′,*N*′′-di-coumaroylspermidine (CoCoS). Structural rearrangements during in source ionization and fragmentation did not allow for unequivocal assignment of the phenylpropanoid residues to the different *N* positions of putrescine and spermidine backbones. CAT: *M. sexta* caterpillar-attacked leaves.

**Figure 7:**
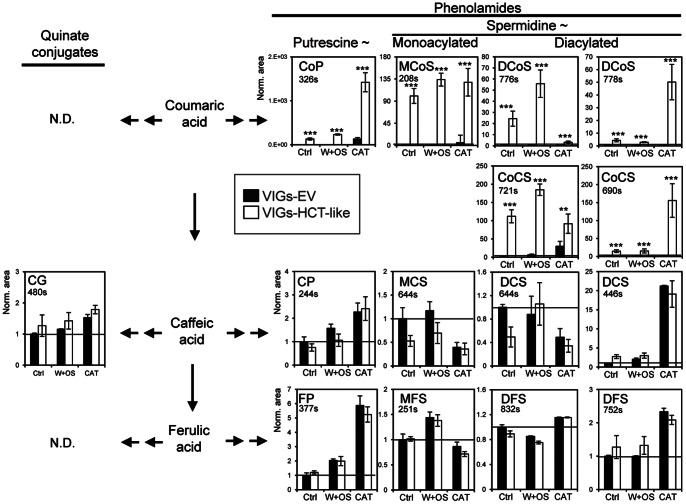
*HCT-LIKE*-silencing changes levels of selected phenolamides and quinate conjugates. Bar charts summarize relative intensities of selected coumaroyl-, caffeoyl- and feruloyl-based mono-acylated putrescine, mono- and di-acylated spermidine metabolites and chlorogenic acids. No robust *m/z* signals associated with coumaroyl- and feruloyl- containing quinate conjugates were detected. The results are consistent with a strong diversion of coumaroyl-coA units into coumaroyl-based PA production. Interestingly, the two herbivory elicitation treatments (caterpillar attack and OS elicitation) elicited isomer-specific responses for most of the PA sub-classes. For example, the intensity of some isomers (DCoS at 778 s) was strongly and specifically amplified by direct insect feeding. (** P<0.01, *** P<0.0001, Student's *t*-test). Ctrl: uninduced control leaves; W+OS: mechanically wounded leaves treated with *M. sexta* oral secretions; CAT: *M. sexta* caterpillar-attacked leaves.

We identified (File S2) several *N*-coumaroylputrescine (CoP, C_13_H_19_N_2_O_2_^+^) isomers, *N*′,*N*′′-dicoumaroylspermidine (CoCoS, C_29_H_32_N_3_O^+^), *N*′,*N*′′-caffeoyl,coumaroylspermidine (CoCS, C_29_H_32_N_3_O_2_^+^) and their monohydrated versions that strongly increased after direct or simulated herbivory of VIGs-HCT-LIKE leaves. Structural rearrangements during in-source ionization and fragmentation did not allow for the unequivocal assignments of the phenylpropanoid residues to the different *N* positions of the putrescine and spermidine backbones. Coumaroylshikimate, the product of HCT activity, could not be detected in VIGs-HCT-LIKE as well as in VIGs-EV leaves, probably due to its rapid turnover. Figure 6 shows the computed traces for some of the most highly up-regulated metabolites, confirming that the production of many of these coumaroyl-based metabolites is specifically activated after *HCT-LIKE*-silencing. As is apparent in Figure 7, *HCT-LIKE*-silencing resulted in a clear diversion of activated *p*-coumaric acid units towards the accumulation of PA, but not towards quinate conjugates. This results most likely from the MYB8-mediated prioritization of the phenolic flux towards PA formation during insect feeding in *N. attenuata* plants. Consistent with this perspective, silencing MYB8 results in the formation of quinate conjugates, which are otherwise absent or in much lower concentrations in WT plants [9]. Interestingly, the fact that isomers of coumaroyl-PA metabolites responded differently to the different herbivore stress treatments (W+OS vs direct feeding), suggests that the strength of the competition between the lignin production and this part of PA metabolism may be compound- or even isomer-specific (Figure 6). This could be achieved, as discussed in [29], through the independent regulations of substrate-specific and regio-isomer-specific *N*-acyltransferases involved in PA production. We have shown that *AT1* which controls the formation of *N*-caffeoylputrescine and *N*-coumaroylputrescine and is among the most strongly elicited genes during insect feeding is not responsible for *N*-feruloylputrescine production, that is one of the major PAs in *N. attenuata* [9]. Moreover, silencing CV86, an enzyme proposed to act on mono-acylated spermidine has been shown to have isomer-specific consequences on the production of diacylated spermidines [9].

No clear changes were detected in the intensity of caffeoyl- and feruloyl-based PAs which normally account for the main part of the PA spectrum in herbivory-elicited *N. attenuata* leaves (Figure S5). We could only detect reductions in the production of the monohydrated forms of *N*′,*N*′′-dicaffeoylspermidine (Figure S5, C_25_H_32_N_3_O_6_^+^), which suggests that diverted coumaroyl-coA units are not recycled into the phenylpropanoid pathway or metabolized after conjugation for the formation of caffeoyl-/feruloyl-based PAs. Indeed, P450 enzymes that can perform the 3-hydroxylation of coumaroyl rings integrated into quinate and spermidine conjugates have been identify in other plants species [30], [31]; the importance of such enzymes for PA production remains unknown in *N. attenuata*. HCT activity has been shown to act both upstream and downstream of the 3-hydroxylation step of the phenylpropanoid pathway [11], in other words to produce both coumaroylshikimate and caffeoylshikimate derivatives from activated *p*-coumaric acid and caffeic acid units (Figure 1). It was therefore surprising that no diversion, as reflected by increased caffeoyl-based PA levels, was detected after silencing *HCT-LIKE*. This observation argues for a central role of *HCT-LIKE* as a coumaroylshikimate/quinate-, rather than a caffeoylshikimate/quinate-, forming enzyme in *N. attenuata* leaves. No peaks corresponding to coumaroylquinate could be detected in the leaves of VIGs-HCT-LIKE and VIGs-EV plants to evaluate the importance of HCT-LIKE activity as a coumaroylquinate-forming enzyme.

## Conclusion

This study provides new insights into herbivory-induced PA production and metabolic interactions with the lignin-associated metabolic network in which it is embedded [1], [32] (Figure 1). This analysis supports recently developed systems-level views on the lignin perturbation response [27] and uncovers a previously undescribed network of coumaroyl-based PAs for which a strong “metabolic tension” exists with the lignin pathway. The metabolic reconfigurations associated with silenced HCT expression revealed a dramatic enrichment in coumaroyl-containing phenolamides. The magnitude of this metabolic diversion depended on the nature of the herbivory treatment applied, and different PA isomers were differently affected, results not readily explained by a “simple” redirection of coumaroyl-coA but rather from more complex and likely MYB8-prioritized reactions, as, for example, underscored by the lack of evidence for increases in quinate conjugates. Finally, this study highlights the value of combining gene-silencing and metabolomics approaches in unraveling the clearly complex interplay among herbivory and developmentally-controlled metabolic responses.

## Supporting Information

Figure S1 **Alignment of** ***N. attenuata*** **HCT-LIKE (HCT-like, NaAT2) deduced protein sequence and highly similar HCT proteins with hydroxycinnamoyl-CoA: shikimate/quinate hydroxycinnamoyltransferase activity from** ***N. tabacum*** **(NtHCT; CAD47830) from** ***N. benthamiana*** **(NbHCT; CAD88491, gene fragment).**
(TIF)

Figure S2 **Design of the VIGs-** ***HCT-LIKE*** **construct and** ***HCT-LIKE*** **gene-silencing efficiency.** (**A**) *Nicotiana attenuata* HCT-LIKE fragment (167 bp) was amplified by PCR using the primer pair shown in grey. The amplified fragment was cloned into the *Bam*HI-*Sal*I sites of the polylinker in the pTV00 vector to obtain pTV-HCT-LIKE. A pTV00 plasmid without insert (empty vector; EV) was used as a negative control in the experiments. To rule out the possibility of off-target silencing, the HCT-like DNA fragment used for VIGs was blasted against a full transcriptome database obtained by 454-sequencing to ensure that this fragment did not have a sequence identity of more than 22 nt with other genes. (**B**) Silencing efficiency. RNA extraction and cDNA synthesis was followed by qRT-PCR analysis with a primer pair designed outside the VIGS silencing region. Elongation factor (EF)-1α gene from tobacco was used for normalization of transcript levels. Like for many other genes, the responsiveness of *HCT-like* expression to the W+OS elicitation (Wound; W + *Manduca sexta* oral secretions; OS) decreases while plants elongate. Therefore, unlike in rosette-stage plants (Figure 2), no induction by the W+OS treatment of *HCT-like* expression was observed in elongated VIGs plants. Most important, The *HCT-LIKE* gene was silenced efficiently, both in control and W+OS-induced plant tissues.
(TIF)

Figure S3 **Principal component analysis (PCA) of metabolic alterations detected in leaves of** ***HCT-LIKE*** **-silenced** ***Nicotiana attenuata*** **plants.** PCA is an unsupervised method identifying principal components that best explain the variance in a data set without referring to class labels. The PCA analysis of metabolites profiles of VIGs-HCT-LIKE and VIGs-EV reveals the herbivory- and HCT-LIKE-specific demarcations of the sample population.
(TIF)

Figure S4 **Volcano plot and Venn diagram representations of significant changes in the** ***Nicotiana attenuata*** **metabolome resulting from** ***HCT-LIKE*** **silencing.** (**A**) Volcano plot representations of differentially regulated *m/z* features in VIGs-HCT-LIKE leaves compared to in VIGs-EV after direct herbivory and simulated herbivory by W+OS elicitation. UHPLC-ESI/TOFMS raw data files from the analysis of methanol-water extracts were pre-processed with the XCMS package. Up or down regulation was assigned to *m/z* features increasing or decreasing in VIGs-HCT-LIKE compared to in VIGs-EV plants with a fold change above 1.5 and P value below 0.05 (unpaired t-test on log_2_-transformed data) (**B**) Venn diagram showing the number of overlapping and non-overlapping differentially regulated *m/z* features between directly attacked leaves and those for which insect feeding was simulated by W+OS elicitation. W+OS: mechanically wounded leaves treated with *M. sexta* oral secretions.
(TIF)

Figure S5 **Silencing** ***HCT-LIKE*** **in** ***Nicotiana attenuata*** **has little effect on induced levels of caffeoyl- and feruloyl-containing metabolites.** Representative ion chromatograms (n = 5) calculated for caffeoyl- (**A**, *m/z* 163.04, Ca) and feruloyl- (**B**, *m/z* 177.05, F) ion moieties generated by in-source fragmentation during analysis by UHPLC-ESI/TOFMS of methanol-water extracts of the *Manduca sexta*-attacked leaves from VIGs-*HCT-LIKE* and VIGs-EV. Leaves of WT and irMYB8 plants were wounded with a fabric pattern wheel and treated with *M. sexta* oral secretions and harvested 24 h later. Ca and F numbers refer to the major PA summarized in the inserted tables. Asteriks indicate significant changes in the relative intensity of reported metabolites between VIGs-*HCT-LIKE* and VIGs-EV samples. Numbers in the compound name column refer to the different annotation levels defined by the Metabolomics Standard Initiative. Cell shading indicates more than 2-fold up-regulation (up, black; down, grey). Ab, abbreviation; FC, fold-changes (VIGs-*HCT-LIKE*>VIGs-EV); Rt, retention time; W+OS: mechanically wounded leaves treated with *M. sexta* oral secretions; CAT: *M. sexta* caterpillar-attacked leaves.
(TIF)

File S1 Examples of MS/MS spectra measured for phenolamides.
(XLSX)

File S2 Complete peak matrix and statistical results.
(XLSX)

## References

[1] Berenbaum MR, Zangerl AR (2008) Facing the future of plant-insect interaction research: le retour à la “raison d'être”. Plant Physiol 146: 804–811. 18316633 10.1104/pp.107.113472 PMC2259083

[2] Pichersky E, Lewinsohn E (2011) Convergent evolution in plant specialized metabolism. Annu Rev Plant Biol 62: 549–566. 21275647 10.1146/annurev-arplant-042110-103814

[3] Fraenkel G (1959) The raison d'être of secondary plant substances. Science: 1466–1470. 10.1126/science.129.3361.1466 13658975

[4] Blechert S, Brodschelm W, Holder S, Kammerer L, Kutchan TM, et al (1995) The octadecanoic pathway: signal molecules for the regulation of secondary pathways. Proc Natl Acad Sci U S A 92: 4099–4105. 7753776 10.1073/pnas.92.10.4099 PMC41893

[5] Mueller MJ, Brodschelm W, Spannagl E, Zenk MH (1993) Signaling in the elicitation process is mediated through the octadecanoid pathway leading to jasmonic acid. Proc Natl Acad Sci U S A 90: 7490–7494. 11607420 10.1073/pnas.90.16.7490 PMC47167

[6] Kaur H, Heinzel N, Schottner M, Baldwin IT, Galis I (2010) R2R3-NaMYB8 regulates the accumulation of phenylpropanoid-polyamine conjugates, which are essential for local and systemic defense against insect herbivores in *Nicotiana attenuata* . Plant Physiol 152: 1731–1747. 20089770 10.1104/pp.109.151738 PMC2832263

[7] Steppuhn A, Gase K, Krock B, Halitschke R, Baldwin IT (2004) Nicotine's defensive function in nature. PLoS Biol 2: E217. 15314646 10.1371/journal.pbio.0020217 PMC509292

[8] Wu JQ, Baldwin IT (2010) New insights into plant responses to the attack from insect herbivores. Annu Rev Genet 44: 1–24. 20649414 10.1146/annurev-genet-102209-163500

[9] Onkokesung N, Gaquerel E, Kotkar H, Kaur H, Baldwin IT, et al (2012) MYB8 controls inducible phenolamide levels by activating three novel hydroxycinnamoyl-coenzyme A:polyamine transferases in *Nicotiana attenuata* . Plant Physiol 158: 389–407. 22082505 10.1104/pp.111.187229 PMC3252090

[10] Hoffmann L, Besseau S, Geoffroy P, Ritzenthaler C, Meyer D, et al (2004) Silencing of hydroxycinnamoyl-coenzyme A shikimate/quinate hydroxycinnamoyltransferase affects phenylpropanoid biosynthesis. Plant Cell 16: 1446–1465. 15161961 10.1105/tpc.020297 PMC490038

[11] Hoffmann L, Maury S, Martz F, Geoffroy P, Legrand M (2003) Purification, cloning, and properties of an acyltransferase controlling shikimate and quinate ester intermediates in phenylpropanoid metabolism. J Biol Chem 278: 95–103. 12381722 10.1074/jbc.M209362200

[12] Kang JH, Wang L, Giri A, Baldwin IT (2006) Silencing threonine deaminase and JAR4 in *Nicotiana attenuata* impairs jasmonic acid-isoleucine-mediated defenses against *Manduca sexta* . Plant Cell 18: 3303–3320. 17085687 10.1105/tpc.106.041103 PMC1693959

[13] Steppuhn A, Gaquerel E, Baldwin IT (2010) The two alpha-dox genes of *Nicotiana attenuata*: overlapping but distinct functions in development and stress responses. BMC Plant Biol 10: 171. 20701756 10.1186/1471-2229-10-171 PMC3017789

[14] Tautenhahn R, Bottcher C, Neumann S (2008) Highly sensitive feature detection for high resolution LC/MS. BMC Bioinformatics 9: 504. 19040729 10.1186/1471-2105-9-504 PMC2639432

[15] Gaquerel E, Heiling S, Schoettner M, Zurek G, Baldwin IT (2010) Development and validation of a liquid chromatography-electrospray ionization-time-of-flight mass spectrometry method for induced changes in *Nicotiana attenuata* leaves during simulated herbivory. J Agric Food Chem 58: 9418–9427. 20701244 10.1021/jf1017737

[16] Sansone SA, Fan T, Goodacre R, Griffin JL, Hardy NW, et al (2007) The metabolomics standards initiative. Nat Biotechnol 25: 846–848. 17687353 10.1038/nbt0807-846b

[17] Xia JG, Wishart DS (2011) Web-based inference of biological patterns, functions and pathways from metabolomic data using MetaboAnalyst. Nature Protocols 6: 743–760. 21637195 10.1038/nprot.2011.319

[18] Boerjan W, Ralph J, Baucher M (2003) Lignin biosynthesis. Annu Rev Plant Biol 54: 519–546. 14503002 10.1146/annurev.arplant.54.031902.134938

[19] Obrien TP, Feder N, Mccully ME (1964) Polychromatic staining of plant cell walls by toluidine blue O. Protoplasma 59: 368–&.

[20] Besseau S, Hoffmann L, Geoffroy P, Lapierre C, Pollet B, et al (2007) Flavonoid accumulation in Arabidopsis repressed in lignin synthesis affects auxin transport and plant growth. Plant Cell 19: 148–162. 17237352 10.1105/tpc.106.044495 PMC1820963

[21] Li X, Bonawitz ND, Weng JK, Chapple C (2010) The growth reduction associated with repressed lignin biosynthesis in *Arabidopsis thalian*a is independent of flavonoids. Plant Cell 22: 1620–1632. 20511296 10.1105/tpc.110.074161 PMC2899864

[22] Mitra S, Baldwin IT (2008) Independently silencing two photosynthetic proteins in *Nicotiana attenuata* has different effects on herbivore resistance. Plant Physiol 148: 1128–1138. 18723666 10.1104/pp.108.124354 PMC2556805

[23] Diezel C, Kessler D, Baldwin IT (2011) Pithy protection: *Nicotiana attenuata*'s jasmonic acid-mediated defenses are required to resist stem-boring weevil larvae. Plant Physiol 155: 1936–1946. 21300916 10.1104/pp.110.170936 PMC3091081

[24] Abbott JC, Barakate A, Pincon G, Legrand M, Lapierre C, et al (2002) Simultaneous suppression of multiple genes by single transgenes. Down-regulation of three unrelated lignin biosynthetic genes in tobacco. Plant Physiol 128: 844–853. 11891241 10.1104/pp.010698 PMC152198

[25] Goujon T, Sibout R, Pollet B, Maba B, Nussaume L, et al (2003) A new Arabidopsis thaliana mutant deficient in the expression of O-methyltransferase impacts lignins and sinapoyl esters. Plant Mol Biol 51: 973–989. 12777055 10.1023/a:1023022825098

[26] Shadle G, Chen F, Srinivasa Reddy MS, Jackson L, Nakashima J, et al (2007) Down-regulation of hydroxycinnamoyl CoA: shikimate hydroxycinnamoyl transferase in transgenic alfalfa affects lignification, development and forage quality. Phytochemistry 68: 1521–1529. 17466347 10.1016/j.phytochem.2007.03.022

[27] Vanholme R, Storme V, Vanholme B, Sundin L, Christensen JH, et al.. (2012) A Systems biology view of responses to lignin biosynthesis perturbations in Arabidopsis. Plant Cell. 10.1105/tpc.112.102574 PMC3480285 23012438

[28] Keinanen M, Oldham NJ, Baldwin IT (2001) Rapid HPLC screening of jasmonate-induced increases in tobacco alkaloids, phenolics, and diterpene glycosides in *Nicotiana attenuata* . J Agric Food Chem 49: 3553–3558. 11513627 10.1021/jf010200+

[29] Bassard JE, Ullmann P, Bernier F, Werck-Reichhart D (2010) Phenolamides: bridging polyamines to the phenolic metabolism. Phytochemistry 71: 1808–1824. 20800856 10.1016/j.phytochem.2010.08.003

[30] Matsuno M, Compagnon V, Schoch GA, Schmitt M, Debayle D, et al (2009) Evolution of a novel phenolic pathway for pollen development. Science 325: 1688–1692. 19779199 10.1126/science.1174095

[31] Schoch G, Goepfert S, Morant M, Hehn A, Meyer D, et al (2001) CYP98A3 from *Arabidopsis thaliana* is a 3′-hydroxylase of phenolic esters, a missing link in the phenylpropanoid pathway. J Biol Chem 276: 36566–36574. 11429408 10.1074/jbc.M104047200

[32] Galis I, Simek P, Narisawa T, Sasaki M, Horiguchi T, et al (2006) A novel R2R3 MYB transcription factor NtMYBJS1 is a methyl jasmonate-dependent regulator of phenylpropanoid-conjugate biosynthesis in tobacco. Plant J 46: 573–592. 16640595 10.1111/j.1365-313X.2006.02719.x

