# Supplementary figures and images for: Silencing an N-Acyltransferase-Like Involved in Lignin Biosynthesis in Nicotiana attenuata Dramatically Alters Herbivory-Induced Phenolamide Metabolism
Source: PLoS One. 2013 May 21;8(5):e62336. doi: 10.1371/journal.pone.0062336 (PMC3660383; doi:10.1371/journal.pone.0062336)

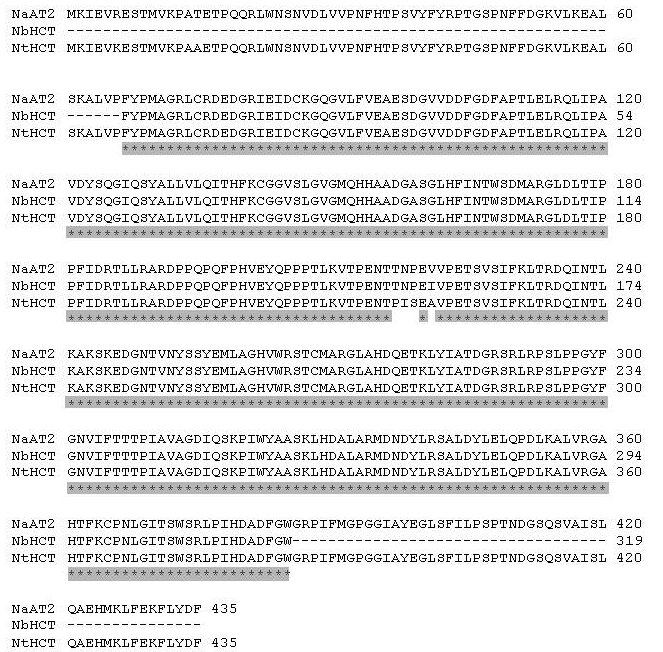

Supplement: Figure S1 — Alignment of N. attenuata HCT-LIKE (HCT-like, NaAT2) deduced protein sequence and highly similar HCT proteins with hydroxycinnamoyl-CoA: shikimate/quinate hydroxycinnamoyltransferase activity from N. tabacum (NtHCT; CAD47830) from N. benthamiana (NbHCT; CAD88491, gene fragment). (TIF) [file pone.0062336.s001.tif]

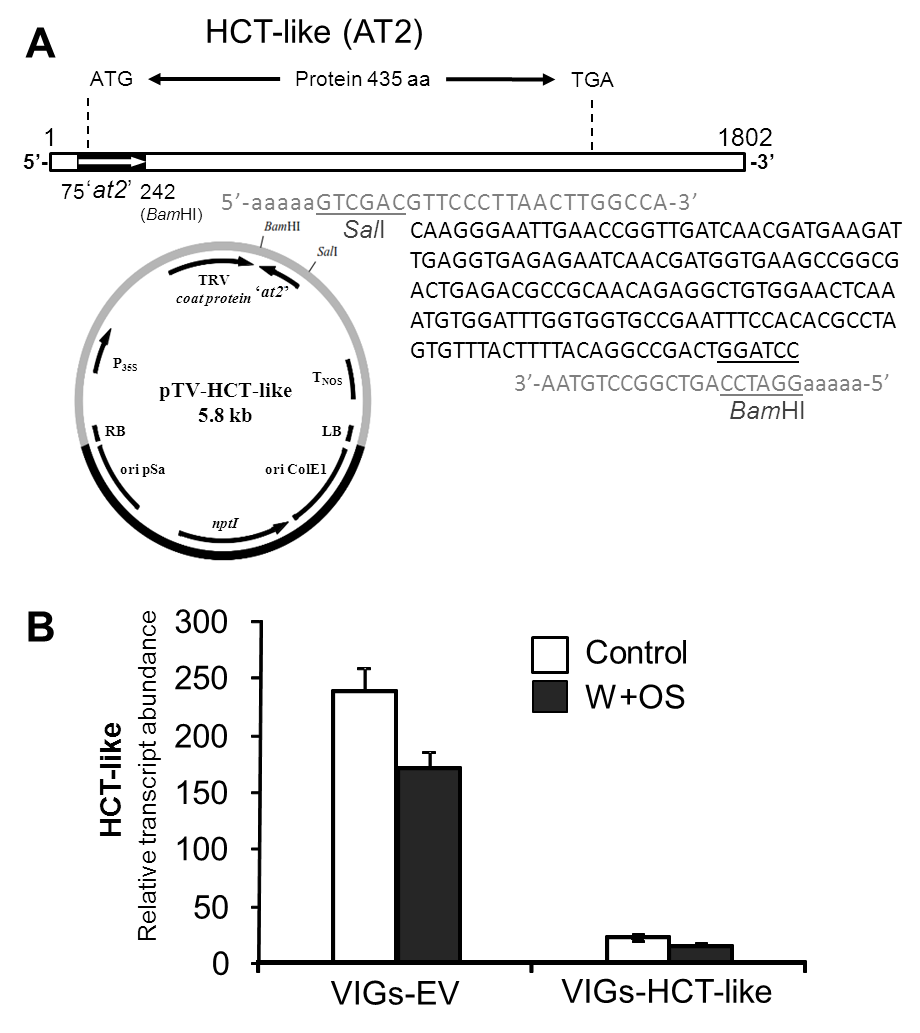

Supplement: Figure S2 — Design of the VIGs- HCT-LIKE construct and HCT-LIKE gene-silencing efficiency. (A) Nicotiana attenuata HCT-LIKE fragment (167 bp) was amplified by PCR using the primer pair shown in grey. The amplified fragment was cloned into the BamHI-SalI sites of the polylinker in the pTV00 vector to obtain pTV-HCT-LIKE. A pTV00 plasmid without insert (empty vector; EV) was used as a negative control in the experiments. To rule out the possibility of off-target silencing, the HCT-like DNA fragment used for VIGs was blasted against a full transcriptome database obtained by 454-sequencing to ensure that this fragment did not have a sequence identity of more than 22 nt with other genes. (B) Silencing efficiency. RNA extraction and cDNA synthesis was followed by qRT-PCR analysis with a primer pair designed outside the VIGS silencing region. Elongation factor (EF)-1α gene from tobacco was used for normalization of transcript levels. Like for many other genes, the responsiveness of HCT-like expression to the W+OS elicitation (Wound; W + Manduca sexta oral secretions; OS) decreases while plants elongate. Therefore, unlike in rosette-stage plants (Figure 2), no induction by the W+OS treatment of HCT-like expression was observed in elongated VIGs plants. Most important, The HCT-LIKE gene was silenced efficiently, both in control and W+OS-induced plant tissues. (TIF) [file pone.0062336.s002.tif]

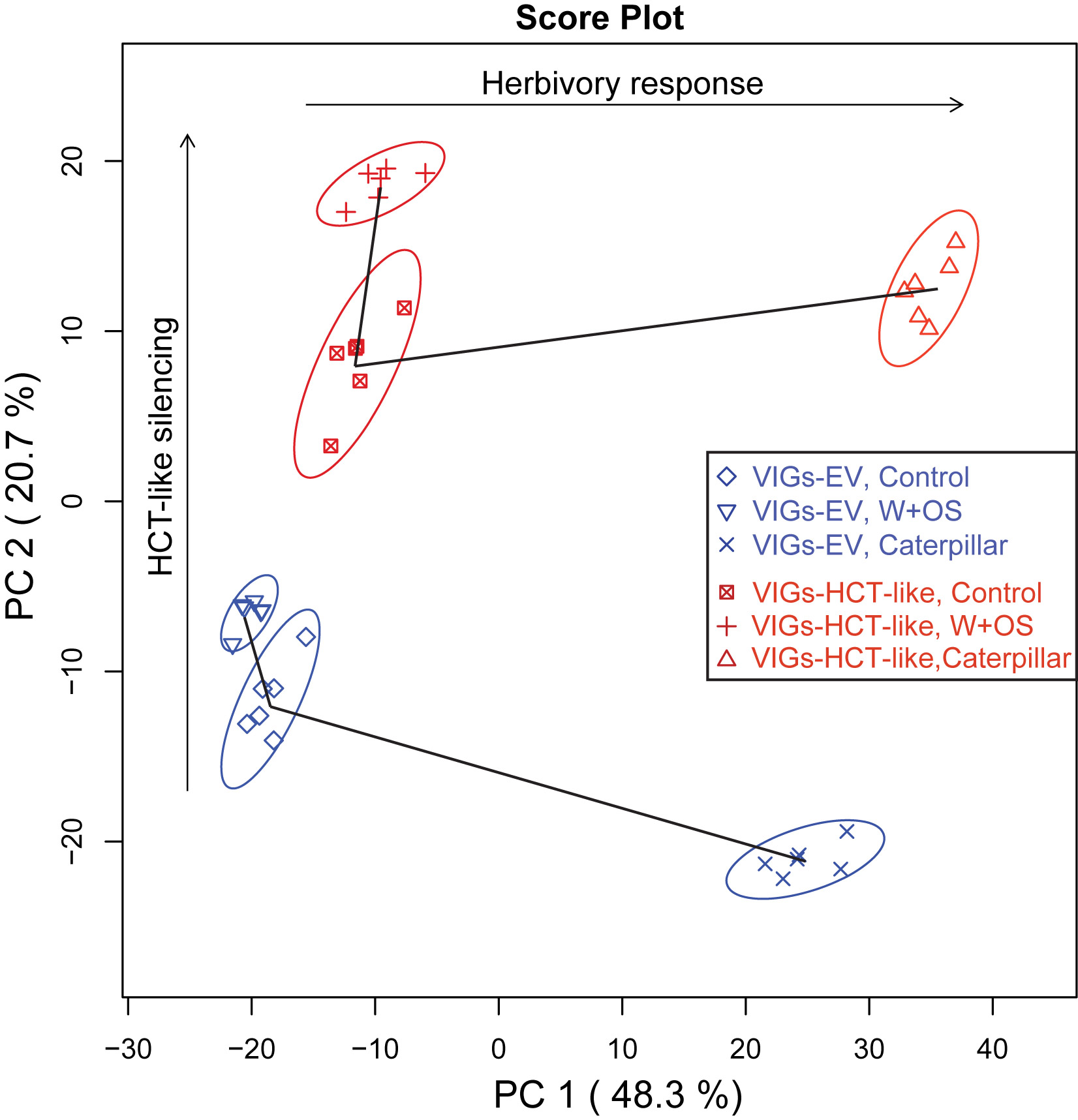

Supplement: Figure S3 — Principal component analysis (PCA) of metabolic alterations detected in leaves of HCT-LIKE -silenced Nicotiana attenuata plants. PCA is an unsupervised method identifying principal components that best explain the variance in a data set without referring to class labels. The PCA analysis of metabolites profiles of VIGs-HCT-LIKE and VIGs-EV reveals the herbivory- and HCT-LIKE-specific demarcations of the sample population. (TIF) [file pone.0062336.s003.tif]

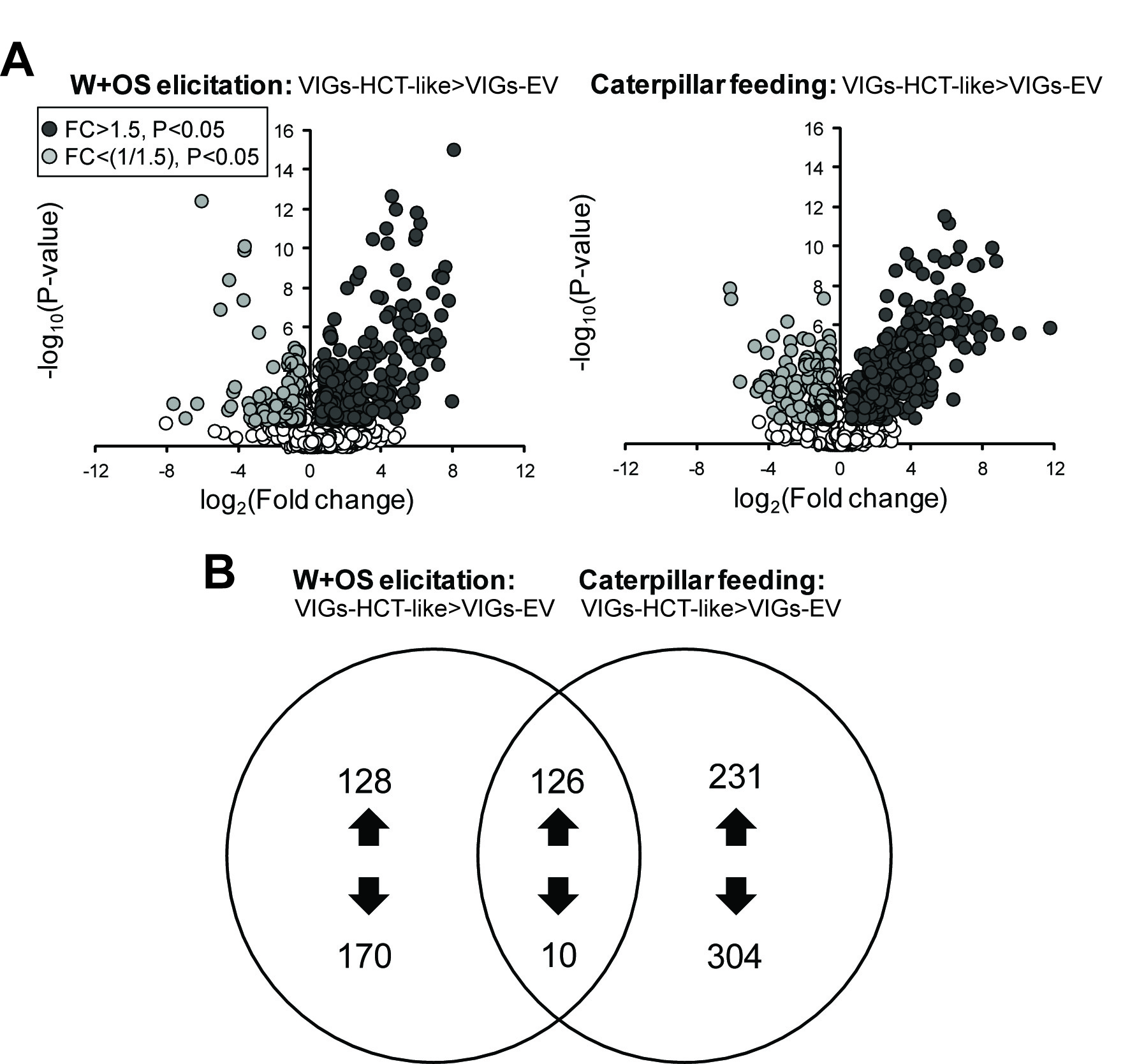

Supplement: Figure S4 — Volcano plot and Venn diagram representations of significant changes in the Nicotiana attenuata metabolome resulting from HCT-LIKE silencing. (A) Volcano plot representations of differentially regulated m/z features in VIGs-HCT-LIKE leaves compared to in VIGs-EV after direct herbivory and simulated herbivory by W+OS elicitation. UHPLC-ESI/TOFMS raw data files from the analysis of methanol-water extracts were pre-processed with the XCMS package. Up or down regulation was assigned to m/z features increasing or decreasing in VIGs-HCT-LIKE compared to in VIGs-EV plants with a fold change above 1.5 and P value below 0.05 (unpaired t-test on log2-transformed data) (B) Venn diagram showing the number of overlapping and non-overlapping differentially regulated m/z features between directly attacked leaves and those for which insect feeding was simulated by W+OS elicitation. W+OS: mechanically wounded leaves treated with M. sexta oral secretions. (TIF) [file pone.0062336.s004.tif]

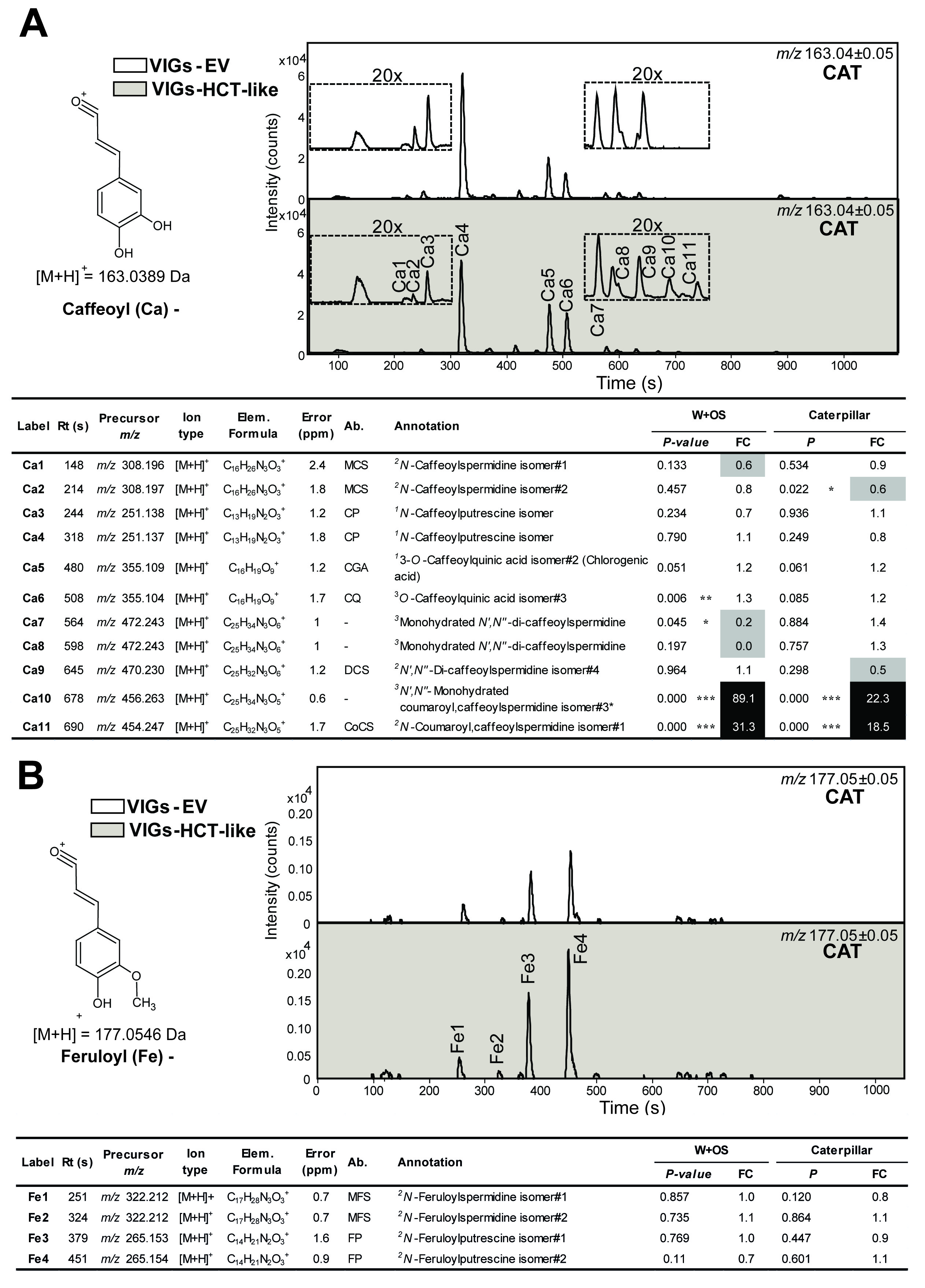

Supplement: Figure S5 — Silencing HCT-LIKE in Nicotiana attenuata has little effect on induced levels of caffeoyl- and feruloyl-containing metabolites. Representative ion chromatograms (n = 5) calculated for caffeoyl- (A, m/z 163.04, Ca) and feruloyl- (B, m/z 177.05, F) ion moieties generated by in-source fragmentation during analysis by UHPLC-ESI/TOFMS of methanol-water extracts of the Manduca sexta-attacked leaves from VIGs-HCT-LIKE and VIGs-EV. Leaves of WT and irMYB8 plants were wounded with a fabric pattern wheel and treated with M. sexta oral secretions and harvested 24 h later. Ca and F numbers refer to the major PA summarized in the inserted tables. Asteriks indicate significant changes in the relative intensity of reported metabolites between VIGs-HCT-LIKE and VIGs-EV samples. Numbers in the compound name column refer to the different annotation levels defined by the Metabolomics Standard Initiative. Cell shading indicates more than 2-fold up-regulation (up, black; down, grey). Ab, abbreviation; FC, fold-changes (VIGs-HCT-LIKE>VIGs-EV); Rt, retention time; W+OS: mechanically wounded leaves treated with M. sexta oral secretions; CAT: M. sexta caterpillar-attacked leaves. (TIF) [file pone.0062336.s005.tif]
